# Deciphering visceral instincts: a scientific quest to unravel food choices from molecules to mind

**DOI:** 10.1101/gad.352279.124

**Published:** 2024

**Authors:** Emily Alway, Naama Reicher, Diego V. Bohórquez

**Affiliations:** 1 Laboratory of Gut Brain Neurobiology, Duke University, Durham, North Carolina 27710; USA;; 2 Department of Medicine, Duke University, Durham, North Carolina 27710; USA;; 3 Department of Neurobiology, Duke University, Durham, North Carolina 27710; USA;; 4 Department of Pathology, Duke University, Durham, North Carolina 27710; USA;; 5 Department of Cell Biology, Duke University, Durham, North Carolina 27710; USA;; 6 Duke Institute for Brain Sciences, Duke University, Durham, North Carolina 27710; USA

**Keywords:** brain–body, physiology, symposium

## Abstract

The study of biological mechanisms, while crucial, cannot fully explain complex phenomena like the instinct to eat. The mind–body connection, as exemplified by the concept of “voodoo death,” highlights the profound influence of belief and cultural context on physiology. Indigenous knowledge systems further emphasize the interconnectedness of humans with their environment. Recent discoveries in gut–brain communication reveal the intricate neural circuits that drive our visceral desires, but a holistic approach that integrates both physiological mechanisms and the subjective experience of life, informed by diverse cultural perspectives, will be essential to truly understand what it means to be alive.

## Abstract (precis)

In this Outlook, Alway et al. use a holistic perspective to understand the complex interplay of biological, psychological, cultural, and environmental factors that underlie human behavior, particularly the act of eating and our relationship with food.

“We are part of the Earth, not apart from it.”

— Joseph M. Marshall, Ph.D.

*To You We Shall Return: Lessons about Our Planet from the Lakota*

Machines are built from parts, each programmed to perform a specific function. Their capabilities are predetermined by the sum of their engineered components. Humans, however, are made of food. We are not just the nutrients that sustain us but the transformation of energy into feelings and instincts—a unique property of being alive. But how does what we eat become who we are?

Our scientific understanding of life has evolved alongside our scientific approach, much like a grand feast with many courses. It began with early inquiries into the subjective experience of self, the “anima,” a concept that captivated philosophers and scientists alike for centuries. However, the rise of Newtonian physics in the 18th century, with its emphasis on objective, measurable phenomena, ushered in a new era of reductionism (Rosen 1985). Life, like all natural phenomena, was to be dissected and understood through universal laws, reducing the complexities of living organisms to the interactions of their fundamental building blocks.

This shift toward reductionism, championed by figures like Erwin Schrödinger, catalyzed the molecular revolution in biology. Schrödinger's exploration of “*What is Life?*” (Schrödinger 1956) and his concept of the “aperiodic crystal” paved the way for the discovery of DNA, the molecule that carries the genetic instructions for life. Since then, the study of life has largely been reduced to the study of molecules like nucleic acids, their interactions, and the mechanisms by which they orchestrate the complex processes within cells.

With this molecular approach, we have achieved extraordinary feats, uncovering the intricate mechanisms that govern physiological processes. We can now describe how cells respond to molecules, electricity, light, or sound and have identified the entangled signaling pathways that underlie these responses. However, as our understanding of these mechanisms deepens, a critical question arises: Can the rich and complex experience of living—and specifically the visceral instinct to eat—be fully captured by this reductionist framework?

Consider the phenomenon of “voodoo death,” a term coined by physiologist Walter B. Cannon in 1942 (Cannon 1942). Cannon described cases in which seemingly healthy individuals died suddenly after being cursed or believing they were doomed. He hypothesized that extreme fear and emotional distress could trigger a cascade of physiological responses, leading to fatal consequences. Similarly to the “placebo effect,” this concept, while initially met with skepticism, highlighted the powerful influence of the mind on the body and challenged the prevailing mechanistic view of life. It echoed the ancient wisdom found in both ethnobotany and Indigenous knowledge systems, which emphasized the profound connection between mind, body, and environment (Schultes and von Reis 1995; Chen et al. 2024).

The ritual practice of zombification in Haiti also serves as a chilling illustration of this intricate mind–body connection. Although the neurotoxin tetrodotoxin plays a role in inducing a death-like state, it is the cultural context and the victim's belief in the sorcerer's power that truly facilitate the transformation. The psychological trauma, amplified by psychoactive substances, reinforces the perceived loss of free will, further blurring the lines between the physical and the psychological (Del Guercio 2017). This complex interplay resonates with the concept of “voodoo death,” where the power of suggestion and belief can have devastating physiological consequences.

Ethnobotany, a field pioneered by figures like Richard Evans Schultes, reveals how Indigenous cultures have long recognized the interconnectedness of humans and the natural world through the lens of food and sustenance (Schultes and von Reis 1995). Schultes’ extensive fieldwork in the Amazon rainforest led to the discovery of numerous plant-based medicines and rituals, demonstrating the ancestral knowledge that Indigenous communities possess about the natural world. This knowledge, often rooted in deep cultural and spiritual traditions, emphasizes the holistic nature of life, encompassing not just the physical but also the mental, emotional, and spiritual dimensions of the act of eating.

In “*Native Science: Natural Laws of Interdependence*,” Gregory Cajete emphasizes that Indigenous knowledge systems view humans as inseparable from the natural world, with a deep understanding of the interdependence of all living things through the sharing of sustenance (Cajete 2000). This perspective is in contrast to the Western scientific tradition, which often emphasizes separation and objectivity. Integrating these diverse ways of knowing can offer a more comprehensive understanding of life, particularly in the realm of food and eating.

Our understanding of these sensory experiences is further expanded in Ed Yong's “*An Immense World*” (Yong 2022). From the echolocation of bats used to locate ripe fruits to the ultraviolet vision of bees guiding them toward nectar-rich flowers, animals perceive the world—and their food—through a multitude of senses that exceed our own capabilities. This diversity of sensory experiences highlights the vastness of the natural world and our interaction with it through food, a complexity that cannot be fully captured by ligands binding to receptors.

Even in the 20th century, scientists like Nicholas Rashevsky were challenging the limitations of a purely mechanistic view of life. Rashevsky argued that life is not simply a collection of gears and levers but an emergent property arising from the complex interactions of its components (Rosen 1985). He proposed a new field, “relational biology,” to study the organizational principles that give rise to life's unique properties—those qualities that make us more than the sum of our parts.

Today, this call for a broader perspective is even more relevant, particularly when we examine our relationship with food. The discovery of specialized gut sensory cells, such as neuropod cells, has illuminated novel neural circuits that underlie our visceral instincts. These cells detect signals within the gut lumen and instantly transmit them to the brain via the vagus nerve, influencing our food choices in real time (Bohórquez et al. 2015; Kaelberer et al. 2018; Buchanan et al. 2022). Furthermore, other gut sensory cells like enterochromaffin cells have been found to play a crucial role in modulating visceral pain and anxiety, emphasizing the intricate connection between the gut and the brain (Bayrer et al. 2023; Chang et al. 2024). These findings, coupled with the discovery of neuropod cells, highlight the intricate dance between the gut, brain, and microbiome, shaping not only our physiological responses but also our overall well-being. It is becoming increasingly evident that the biological mechanisms governing our perception and response to stimuli are deeply intertwined, further emphasizing the need for a holistic approach to understanding life (Fig. 1; Ran et al. 2022).

**Figure 1.**
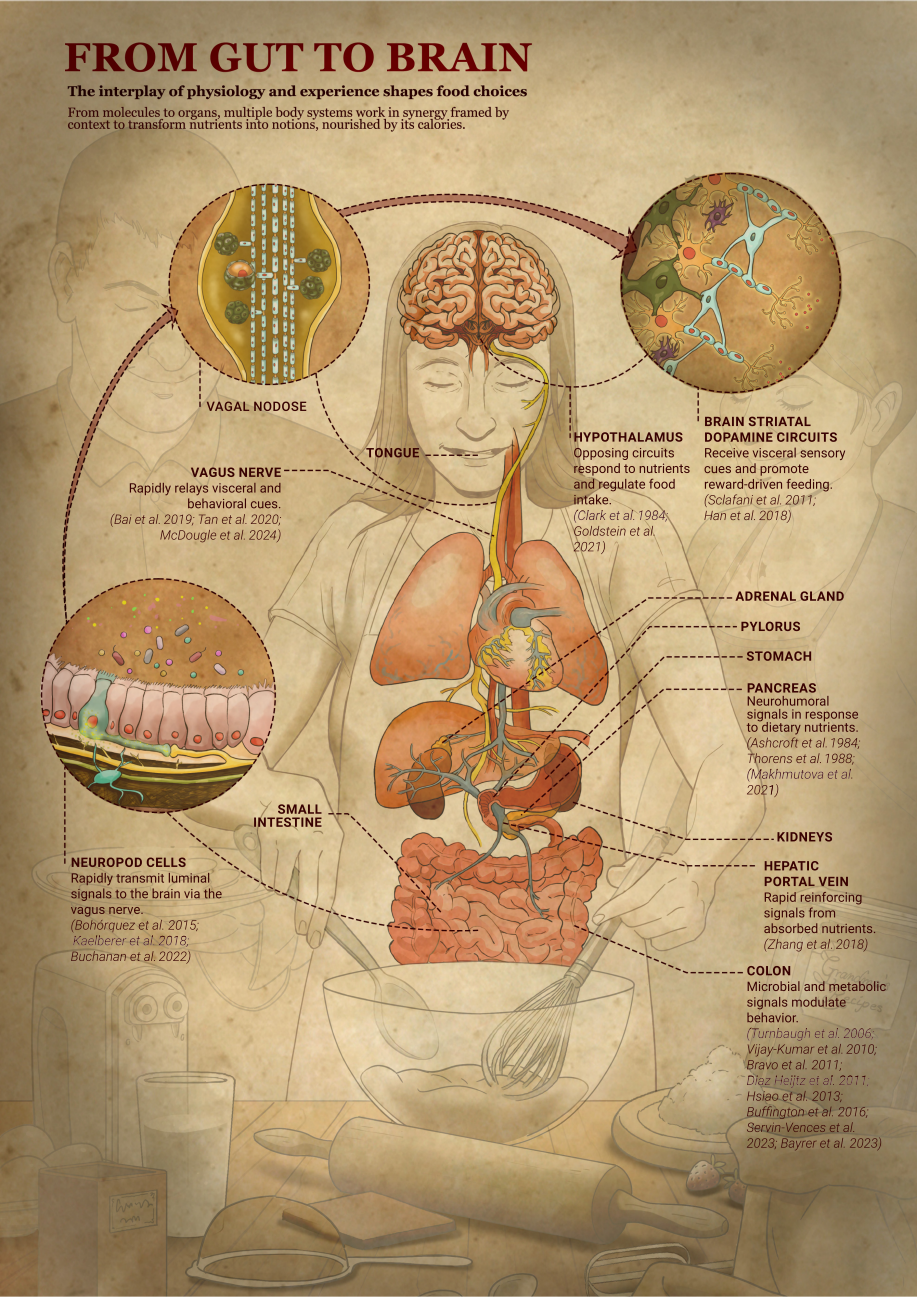
From gut to brain. The interplay of physiology and experience shapes food choices.

Although these mechanistic insights into visceral signaling are at the core of defining interoceptive physiology, they do not fully capture the subjective experience of behavior. Consider the simple act of eating: The joy of baking a birthday cake in the company of friends or the comfort of a warm cookie on a cold day transcends mere neural firing or hormonal release. These experiences are woven from a tapestry of biological, psychological, and cultural threads, mirroring the intricate interplay of factors observed in phenomena like “voodoo death” and zombification. Even the seemingly simple preference for sugar over artificial sweeteners hints at the complex dance between nutrient sensing and gut–brain communication that shapes our dietary choices (Tan et al. 2020; Buchanan et al. 2022). Our gut instincts drive us to consume food that then becomes a part of who we are.

In the era of artificial intelligence, this distinction between the objective and subjective becomes even more pronounced. While we can build machines that replicate our functions, we cannot yet program them to experience the world as we do. It is this subjective experience, this capacity for feeling, that makes us human.

As we continue our scientific odyssey of deciphering visceral instincts, it is imperative to recognize the limitations of a purely reductionist approach. We must embrace the complexity, the mystery, and the wonder of our visceral instincts, just as ethnobotany and Indigenous knowledge systems, alongside insights into the animal world, have broadened our understanding of the interconnectedness of life. By integrating our mechanistic understanding with an appreciation for the subjective experience of life and the wisdom of diverse cultures, we can embark on a new chapter in our quest to understand how the body and mind make us feel alive.
